# Vitamin D deficiency associates with susceptibility to tuberculosis in Pakistan, but polymorphisms in *VDR*, *DBP* and *CYP2R1* do not

**DOI:** 10.1186/s12890-016-0240-2

**Published:** 2016-05-10

**Authors:** Kashaf Junaid, Abdul Rehman, David A. Jolliffe, Tahir Saeed, Kristie Wood, Adrian R. Martineau

**Affiliations:** Department of Microbiology and Molecular Genetics, University of the Punjab, Quaid-e-Azam Campus, Lahore, 5400 Pakistan; Centre for Primary Care and Public Health, Barts and The London School of Medicine and Dentistry, Queen Mary University of London, London, E1 2AB UK; Gulab Devi Chest Hospital, Lahore, Pakistan; Genome Centre, Barts and the London School of Medicine and Dentistry, Queen Mary University of London, London, EC1M 6BQ UK

**Keywords:** Tuberculosis, Vitamin D, Vitamin D Receptor, Hypovitaminosis D, Vitamin D binding protein, Vitamin D 25-hydroxylase

## Abstract

**Background:**

Single nucleotide polymorphisms (SNPs) in the genes encoding the vitamin D receptor (*VDR*) and the vitamin D binding protein (*DBP*) have been reported to modify the influence of vitamin D deficiency on susceptibility to active tuberculosis (TB) in the UK, but this phenomenon has not been investigated in settings with a high TB burden. SNPs in *CYP2R1*, which encodes a vitamin D 25-hydroxylase enzyme, are known to influence vitamin D status, but their potential role in determining susceptibility to TB has not previously been investigated in any setting.

**Method:**

We conducted a case–control study in 260 pulmonary TB patients and 112 controls recruited in Lahore, Pakistan. Analyses were conducted to test for main effects of vitamin D status and SNPs in *VDR* (rs731236, rs2228570 and rs1544410), *DBP* (rs7041 and rs4588) and *CYP2R1* (rs2060793, rs10500804 and rs10766197) on susceptibility to TB, and to investigate whether these SNPs modify the association between vitamin D status and disease susceptibility.

**Results:**

Profound vitamin D deficiency (serum 25-hydroxyvitamin D concentration ≤ 20 nmol/L) was common among TB patients (118/260, 45 %), and was independently associated with susceptibility to TB (adjusted odds ratio 1.87, 95 % CI 1.15 to 3.04, *P* = 0.01). However, none of the SNPs investigated associated with susceptibility to TB, either in main effects analysis, or in interaction with vitamin D status.

**Conclusion:**

Profound vitamin D deficiency was common among TB patients in this high-burden setting, and was independently associated with disease susceptibility. However, no statistically significant associations between SNPs in the vitamin D pathway and disease susceptibility was demonstrated.

**Electronic supplementary material:**

The online version of this article (doi:10.1186/s12890-016-0240-2) contains supplementary material, which is available to authorized users.

## Background

Tuberculosis (TB) is a leading global cause of morbidity and mortality, responsible for an estimated 1.5 million deaths in 2014 [1]. The identification of factors which increase disease susceptibility has potential to inform control strategies. Case control studies have previously reported independent associations between vitamin D deficiency and susceptibility to active TB [2]. However, interpretation of such studies is limited by their cross-sectional nature, as they cannot determine whether vitamin D deficiency is a cause or a consequence of active TB. The case for vitamin D deficiency playing a causal role in enhancing disease susceptibility is supported by findings of genetic studies reporting that polymorphisms in the vitamin D receptor (VDR) and the vitamin D binding protein (DBP) genes modify the influence of vitamin D status on susceptibility to active disease in the UK [3, 4]. Only one study is present who have investigated such gene-environment interactions; analogous investigations in high TB burden countries are therefore needed [5]. Moreover, no study has previously investigated whether polymorphisms in the gene encoding the vitamin D hydroxylase enzyme, *CYP2R1,* are associated with susceptibility to TB, either as a main effect, or as a modifier of the effects of vitamin D status. This gene is a plausible candidate for effect modification, as 25-hydroxylation of ‘parent’ vitamin D_3_ (cholecaciferol, synthesized in the skin on exposure to ultra-violet B radiation) to form the major circulating vitamin D metabolite 25-hydroxyvitamin D (25[OH]D) is a key determinant of circulating 25(OH)D concentrations [6].

We therefore conducted a case–control study to determine the influence of vitamin D status and single nucleotide polymorphisms (SNPs) in *VDR*, *DBP* and *CYP2R1* on susceptibility to active TB in Pakistan, a high-burden setting where TB incidence in 2014 was estimated at 270 cases per 100,000 population per year [1]. Having demonstrated an independent association between vitamin D deficiency and susceptibility to active TB in the study population, we proceeded to investigate whether this was modified by SNPs in the vitamin D pathway.

## Methods

### Study design

We conducted a case control study. Cases were patients aged 14–60 years with newly-diagnosed smear-positive pulmonary TB, recruited from the Gulab Devi Chest hospital, Lahore. Patients with any of the following conditions documented in the hospital record were excluded: diabetes mellitus, ischemic heart disease, chronic renal failure, jaundice or seropositivity for hepatitis B surface antigen, Hepatitis C Virus or Human Immunodeficiency Virus. Controls were healthy relatives of TB patients or hospital staff working at the same hospital with no history of previous tuberculosis but exposed to TB cases. Participants who fulfilled eligibility criteria were asked to complete a questionnaire detailing their age, gender and monthly income. Informed consent was taken from all participants before sample collection. The study was approved from the ethical committee of the University of Punjab (Ref No: SBS 873–12) and also from Gulab Devi Chest Hospital, Lahore.

Five mL of blood were drawn from a median cubital vein; 2 mL were transferred into vials containing EDTA and frozen at −20 °C for subsequent DNA extraction, and 3 mL were added to serum vials and sent to the laboratory within two hours of collection, where serum was isolated from clotted blood by centrifugation and stored at −20 °C for subsequent determination of 25(OH)D concentration.

### Serum 25 (OH)D assay

Serum 25(OH)D concentration was determined by ELISA (Immunodiagnostic Systems, Boldon,UK). Calibrators and controls provided with kits were run in duplicate. For the purpose of this study, a patient with 25(OH) vitamin D level equal or less than 20 nmol/L was considered to be vitamin D deficient. Inter-assay CV for serum 25(OH)D assay for our samples was 12.5 %.

### Genotyping

Genomic DNA was extracted from whole blood and quantified using a nanodrop spectrophotometer as previously described [7]. TaqMan allelic probe assays (Applied Biosystems, Foster City, CA, USA) were used to genotype polymorphisms in genes encoding the vitamin D receptor *VDR* [(rs731236 Taq I), (rs2228570 Bsm I); (rs1544410 Fok I)]; the vitamin D 25-hydroxylase *CYP2R1* [(rs2060793, rs10500804, rs10766197)] and the vitamin D binding protein *DBP* [(rs7041) and (rs4588)]. All primers were commercially prepared. Details of primers used in this study has been provided in Additional file 1. The PCR reaction mixture contained 7.5 μL of TaqMan Genotyping master mixture, 0.75 μL of TaqMan SNP (probes), 1 μL of genomic DNA (1–10 ng), and 5.75 μL nuclease free water. Thermal conditions were as follows: initial denaturation at 95 °C for 10 min, 40 cycles were run at 95 °C for 15 seconds (denaturing) followed by 60 °C for 1 min (annealing/extension). PCR plates were read by 7900HT seq detection system ABI prism.

### Sample size and statistical analysis

Sample size was calculated using OpenEpi sample calculator [8] assuming an expected frequency of 57 % vitamin D deficiency in cases and 33 % in controls [9]. Statistical analyses were done with SPSS version 20. Chi square tests were used for univariate analyses exploring potential determinants of vitamin D status and susceptibility to active TB, and binary logistic regression analysis was used for corresponding multivariate analyses. Sub-group analyses were performed to determine whether genetic variation in the vitamin D pathway modified effects of vitamin D status on susceptibility to active TB by repeating primary efficacy analyses with the inclusion of a term for an interaction between vitamin D status and genotype. Odds ratios are presented with 95 % confidence intervals and *P*-values.

## Results

### Participant characteristics

**Fig. 1:**
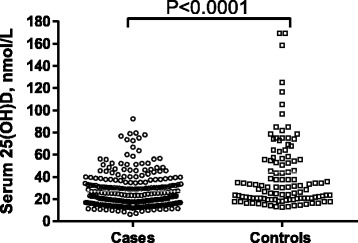
Serum 25-hydroxyvitamin D (25[OH]D) concentrations in TB cases (*n* = 260) and healthy controls (*n* = 112). *P* value from unpaired *t* test comparison of means

A total of 260 cases with smear-positive pulmonary TB and 112 controls were recruited to the study between August 2012 and September 2013. The mean age of cases and controls was 31.6 years (s.d. 10.5 years, range 14–55 years) vs. 30.6 years (s.d. 9.3, range 15–56 years) respectively. Fifty-four percent of cases and 48 % of controls were female. Forty-five percent of cases and 30 % of controls had profound vitamin D deficiency (serum 25[OH]D ≤20 nmol/L), and mean serum 25(OH)D concentration was lower in cases vs. controls (27.3 vs. 43.3 nmol/L respectively, 95 % CI for difference 11.5–22.8, *P* < 0.0001; Fig. 1).

### Determinants of vitamin D status in cases and controls

Table 1 presents determinants of vitamin D status in cases and controls. Vitamin D deficiency was independently associated with female vs. male gender (aOR 2.31, 95 % CI 1.33 to 4.01, *P* = 0.003; aOR 3.67, 95 % CI 1.38 to 9.70, *P* = 0.01) and sampling in October-March vs. April-September (aOR 1.89, 95 % CI 1.14 to 3.16, *P* = 0.01; aOR 2.55, 95 % CI 1.03 to 6.31, *P* = 0.04) in both case and control populations, respectively (Table 1).

**Table 1:** Determinants of vitamin D status in TB cases and controls

		TB cases			Control		
Characteristics		25 (OH) D ≤20 nmol/l	aOR(95 % CI)^a^	P	25(OH) D ≤20 nmol/l	aOR(95 % CI)^a^	P
Age	>30 years	56/124 (45 %)	Ref		17/50 (34 %)	Ref	
	≤30 years	62/136 (46 %)	1.26(0.71-2.32)	0.44	17/62 (27 %)	1.46(0.60-3.56)	0.40
Sex	Male	43/120 (36 %)	Ref	-	11/58 (19 %)	Ref	-
	Female	75/140 (54 %)	2.31(1.33-4.01)	0.003	23/54 (43 %)	3.67(1.38-9.70)	0.01
Marital status	Single	49/114 (43 %)	Ref	-	16/54 (30 %)	Ref	-
	Married	69/146 (46 %)	1.34(0.71-2.55)	0.36	18/58 (31 %)	1.63(0.64-1.43)	0.30
Monthly income, PKR	<10,000	84/177 (47 %)	1.27(0.73-2.20)	0.38	18/62 (29 %)	1.0(0.42-2.40)	0.98
	≥10,000	34/83 (41 %)	Ref	-	16/50 (32 %)	Ref	-
Month of recruitment	April-September	53/137 (39 %)	Ref	-	10/53 (19 %)	Ref	-
	October-March	65/123 (53 %)	1.89(1.14-3.16)	0.01	24/59 (41 %)	2.55(1.03-6.31)	0.04

^a^Odds ratio adjusted for age, sex, marital status, monthly income and month of recruitment. *PKR* Pakistani rupees, 25(OH)D, 25-hydroxyvitamin D

### Association between phenotypic features and susceptibility to active tuberculosis

Table 2 presents results of univariate and multivariate analyses testing for associations between participants’ phenotypic features and susceptibility to active TB. Active TB was independently associated with lower monthly income (aOR 1.68, 95 % CI 1.06 to 2.66, *P* = 0.02) and presence of profound vitamin D deficiency (aOR 1.87, 95 % CI 1.15 to 3.04, *P* = 0.01). The following phenotypic features were not found to associate with susceptibility to active TB: age, sex, marital status or season of recruitment.

**Table 2:** Association between participants phenotypic characteristics and susceptibility to active tuberculosis

		TB cases (*N* = 260)	Control (*N* = 112)	OR (95 % CI)	P	aOR(95 % CI)^a^	P
Age	>30 years	124 (48 %)	50 (45 %)	Ref	-	Ref	-
	≤30 years	136 (52 %)	62 (55 %)	1.01(0.58-1.42)	0.68	1.01(0.61-1.68)	0.94
Sex	Male	120 (46 %)	58 (52 %)	Ref	-	Ref	-
	Female	140 (54 %)	54 (48 %)	1.25(0.80-1.95)	0.31	1.06(0.65-1.75)	0.79
Marital status	Single	114 (44 %)	54 (48 %)	Ref	-	Ref	-
	Married	146 (56 %)	58 (52 %)	1.19(0.76-1.86)	0.43	1.13(0.67-1.90)	0.63
Monthly income, PKR	<10,000	177 (68 %)	62 (55 %)	1.72(1.10-2.71)	0.02	1.68(1.06-2.66)	0.02
	≥10,000	83 (32 %)	50 (45 %)	Ref	-	Ref	-
Season of recruitment	April-September	137 (53 %)	53 (47 %)	1.24(0.79-1.23)	0.34	1.24(0.78-1.96)	0.34
	October-March	123 (47 %)	59 (53 %)	Ref	-	Ref	-
Serum 25(OH)D, nmol/L	≤20 nmol/L	118 (45 %)	34 (30 %)	1.90(1.19-3.05)	0.02	1.87(1.15-3.04)	0.01
	>20 nmol/L	142(55 %)	78(70 %)	Ref	-	Ref	-

^a^Odds ratio adjusted for age, sex, marital status, monthly income, season of recruitment and serum 25(OH)D concentration. PKR, Pakistani rupees; 25(OH)D, 25-hydroxyvitamin D

### Association between vitamin D pathway genotype and susceptibility to active tuberculosis

Table 3 presents results of analyses testing for associations between genetic variation in *VDR, CYP2R1* and *DBP* and susceptibility to active TB, adjusting for monthly income and vitamin D status. No statistically significant association was observed between susceptibility to active TB and any polymorphism investigated.

**Table 3:** Association between vitamin D pathway genotype and susceptibility to active tuberculosis

SNP, gene	Genotype	TB cases, n/N (%)	TB contacts, n/N (%)	aOR (95 % CI)^a^	P
rs7312236, *VDR*	AA	84/230 (37 %)	42/100 (42 %)	Ref	-
	AG/GG	146/230 (63 %)	58/100 (58 %)	1.22(0.75-1.98)	0.42
rs154410, *VDR*	CC	53/235 (23 %)	29/106 (27 %)	Ref	-
	CT/TT	182/235 (77 %)	77/106 (73 %)	1.23(0.71-2.09)	0.45
rs228570, *VDR*	GG	137/235 (59 %)	68/107 (64 %)	Ref	-
	GA/AA	98/235 (41 %)	39/107 (36 %)	1.89(0.80-2.10)	0.28
rs7041, *DBP*	AA	55/232 (24 %)	21/102 (21 %)	Ref	-
	AC/CC	177/232 (76 %)	81/102 (79 %)	0.77(0.43-1.39)	0.39
rs4588, *DBP*	GG	122/234 (52 %)	50/107 (47 %)	Ref	-
	GT/TT	112/234 (48 %)	57/107 (53 %)	0.93(0.58-1.49)	0.77
rs10500804, *CYP2R1*	GG	59/231 (26 %)	22/103 (22 %)	Ref	-
	GT/TT	172/231 (74 %)	81/103 (78 %)	0.83(0.47-1.47)	0.53
rs2060793, *CYP2R1*	GG	112/228 (49 %)	51/101 (50 %)	Ref	-
	AG/AA	116/228 (51 %)	50/101 (50 %)	0.91(0.56-1.48)	0.72
rs10766197, *CYP2R1*	AA	52/232 (23 %)	19/104 (18 %)	Ref	-
	AG/GG	124/232 (77 %)	55/104 (82 %)	0.68(0.37-1.26)	0.23

^a^Odds ratio adjusted for monthly income and vitamin D status, *SNP* single nucleotide polymorphism, *Ref* Referent category, *VDR* Vitamin D receptor, *CYP2R1* Vitamin D 25-hydroxylase, *DBP* Vitamin D binding protein. The total number of samples is less than 260 and 112 in cases and controls respectively due to failed genotyping for some samples

### Association between vitamin D deficiency and host genotype, stratified by vitamin D status

We next proceeded to investigate whether genetic variation in *VDR, DBP* or *CYP2R1* modified the association between vitamin D deficiency and susceptibility reported above. Results of the pertinent sub-group and interaction analyses are presented in Table 4: these revealed no evidence to support the hypothesis that polymorphisms in the vitamin D pathway modify the effect of vitamin D deficiency on risk of active TB.

**Table 4:** Association between vitamin D deficiency and host genotype, stratified by vitamin D status

SNP, gene	Genotype	Serum 25(OH) D ≤20 nmol/l				Serum 25(OH) D >20 nmol/l					
		TB cases	Controls	OR(95 % CI)	P	TB cases	Control	OR(95 % CI)	P	Adjusted ratio of odds ratios^a^	P _interaction_
rs7312236, *VDR*	AA	30/102(29 %)	12/24(50 %)	Ref	-	54/128(42 %)	30/76(40 %)	Ref	-	Ref	-
	AG/GG	72/102(71 %)	12/24(50 %)	2.40(0.96-5.94)	0.054	74/128(58 %)	46/76(60 %)	0.89(0.50-1.59)	0.70	1.65(0.59-4.58)	0.33
rs154410, *VDR*	CC	16/105(15 %)	8/25(32 %)	Ref	-	37/130(29 %)	21/81(26 %)	Ref		Ref	-
	CT/TT	89/105(85 %)	17/25(68 %)	2.61(0.96-7.07)	0.052	93/130(71 %)	60/81(74 %)	0.88(0.47-1.64)	0.68	2.16(0.69-6.75)	0.18
rs228570, *VDR*	GG	60/105(57 %)	13/25(52 %)	Ref	-	77/130(59 %)	55/82(67 %)	Ref		Ref	-
	GA/AA	45/105(43 %)	12/25(48 %)	0.81(0.33-1.94)	0.64	53/130(41 %)	27/82(33 %)	1.40(0.78-2.5)	0.25	0.48(0.17-1.29)	0.14
rs10500804, *CYP2R1*	GG	27/102(26 %)	6/23(26 %)	Ref	-	32/129(25 %)	16/80(20 %)	Ref		Ref	-
	GT/TT	75/102(74 %)	17/23(74 %)	0.98(0.35-2.74)	0.97	97/129(75 %)	64/80(80 %)	0.75(0.38-1.49)	0.42	1.51(0.47-4.8)	0.48
rs2060793, *CYP2R1*	GG	48/104(46 %)	11/22(50 %)	Ref	-	64/124(52 %)	40/79(51 %)	Ref		Ref	-
	AG/AA	56/104(54 %)	11/22(50 %)	1.16(0.46-2.92)	0.74	60/124(48 %)	39/79(49 %)	0.96(0.54-1.69)	0.89	1.02(0.37-2.78)	0.96
rs10766197, *CYP2R1*	AA	23/104(22 %)	4/23(17 %)	Ref	-	29/128(23 %)	15/81(19 %)	Ref		Ref	-
	AG/GG	81/104(78 %)	19/23(83 %)	0.74(0.22-2.39)	0.60	99/128(77 %)	66/81(81 %)	0.77(0.38-1.55)	0.47	1.25(0.35-4.45)	0.72
rs7041, *DBP*	AA	27/104(52 %)	4/23(43.5 %)	Ref	-	26/133(20 %)	19/74(47 %)	Ref		Ref	-
	AC/CC	77/10(35 %)	19/23(43.5 %)	0.60(0.18-1.9)	0.39	107/133(80 %)	55/74(45 %)	1.42(0.72-2.79)	0.30	1.15(0.34-3.88)	0.81
rs4588, *DBP*	GG	55/105(52 %)	10/24(42 %)	Ref	-	68/135(50 %)	38/76(50 %)	Ref	-	Ref	-
	GT/TT	50/105(48 %)	14/24(58 %)	0.64(0.26-1.59)	0.34	67/135(50 %)	38/76(50 %)	0.98(0.56-1.72)	0.95	0.93(0.35-2.48)	0.89

^a^Adjusted for monthly income and vitamin D status, *SNP* single nucleotide polymorphism, *Ref* Referent category; *VDR*, Vitamin D receptor; *CYP2R1*, Vitamin D 25-hydroxylase; *DBP*, Vitamin D binding protein. The total number of samples is less than 260 and 112 in cases and controls respectively due to failed genotyping for some samples

## Discussion

We report that vitamin D deficiency is very common among TB patients in Pakistan, and that it associates independently with susceptibility to active TB. Risk of deficiency was independently associated with sampling in January - March and with female gender in cases and controls alike. However, we found no evidence to suggest that polymorphisms in *CYP2R1*, *VDR* or *DBP* influenced susceptibility to TB in this population, either as main effects, or in interaction with vitamin D deficiency.

Our finding of an independent association between vitamin D deficiency and susceptibility to active TB is in keeping with previous reports from Pakistan [9] and elsewhere [10]. A prospective study in Pakistan has previously reported that vitamin D deficiency precedes onset of active disease [11], and laboratory studies have reported that vitamin D metabolites induce antimycobacterial activity in vitro [12]: taken together, such reports suggest that vitamin D deficiency plays a causal role in increasing susceptibility to active TB. However, cross-sectional studies such as our own cannot distinguish causality from reverse causality, and the possibility remains that active TB may be a cause, as well as a consequence, of vitamin D deficiency.

By contrast, our study did not replicate previous reports of associations between polymorphisms in *VDR* and *DBP* and susceptibility to active TB [4, 13], either as main effects or in interaction with serum 25(OH)D concentration. Neither did we find any such associations for variants in *CYP2R1*. “A recent study in pulmonary TB in a cohort of HIV-infected and HIV exposed uninfected children in South Africa also showed no association between polymorphisms in VDR, DBP and CYP2R1 and a diagnosis of TB [5]. It should be noted, however, that our study was formally powered to detect associations between vitamin D deficiency and susceptibility to active tuberculosis, rather than genetic associations: the strength of previously reported associations is modest, and we may have lacked power to detect weaker associations. Another limitation of this study is that we collected information on a limited set of confounders. Despite adjustment for these, we cannot exclude the possibility that residual confounding may have contributed to the association seen.

Women participants in this study had lower vitamin D status that was in keeping with our previous reports that female gender is a risk factor for vitamin D deficiency; women may experiencing less sun exposure than men, and may also have lower dietary vitamin D intake [14, 15].

## Conclusion

In conclusion, our study reports an independent association between vitamin D deficiency and susceptibility to active tuberculosis in Pakistani adults. However, polymorphisms in *VDR*, *DBP* and *CYP2R1* were not found to associate with susceptibility to disease in this setting.

### Availability of data and materials

Primers used in this study are provided as supplementary information accompanying this paper.

## Additional file

Additional file 1: TaqMan allelic assay primers and probes used in real time PCR. (DOCX 12 kb)
