# Supplementary material for: Vitamin D deficiency associates with susceptibility to tuberculosis in Pakistan, but polymorphisms in VDR, DBP and CYP2R1 do not
Source: BMC Pulm Med. 2016 May 10;16:73. doi: 10.1186/s12890-016-0240-2 (PMC4862192; doi:10.1186/s12890-016-0240-2)
Supplement: Additional file 1: — TaqMan allelic assay primers and probes used in real time PCR. (DOCX 12 kb) [file 12890_2016_240_MOESM1_ESM.docx]

Supplementary Appendix 1:

TaqMan allelic assay primers and probes used in real time PCR

| **SNP ID (assay ID)** | **Gene** | **Primers attached with VIC and FAM Flourescent material** |
| --- | --- | --- |
| rs7041 (C-3133594_30) | VDB (GC) | GCTTTGCCAGTTCCGTGGGTGTGGC**[A/C]**TCAGGCAATTTTGCTTTTAGTCGCT |
| rs4588 (C_8278879-10) | VDB (GC) | CTTGTTAACCAGCTTTGCCAGTTCC**[G/T]**TGGGTGTGGCATCAGGCAATTTTGC |
| rs2060793(C-2958431_10) | CYP2R1 | ATATTGGGCCCACCTGGATAATCCC**[A/G]**ACTCAAAGATCAGCTGATTAGCCCT |
| rs10500804 (C_2958429_10) | CYP2R1 | ACTCCTTAGGAGCAGTGATTTCATC**[G/T]**TTTTCATCTTTGAATCCTTACACAG |
| rs10766197 (C-2958435_10) | CYP2R1 | CCAGTTAATTAGAGATCTTTAAACT**[A/G]**TGCCAAGATACAGAAAGGACCAAGC |
| rs731236 (C-2404008) | VDR | TGGACAGGCGGTCCTGGATGGCCTC**[A/G]**ATCAGCGCGGCGTCCTGCACCCCAG |
| rs2228570 (C-12060045_20) | VDR | GGAAGTGCTGGCCGCCATTGCCTCC**[A/G]**TCCCTGTAAGAACAGCAAGCAGGCC |
| rs1544410 (C_8716062_10) | VDR | GAGCAGAGCCTGAGTATTGGGAATG**[C/T]**GCAGGCCTGTCTGTGGCCCCAGGAA |
